# Sampling multiple life stages significantly increases estimates of marine biodiversity

**DOI:** 10.1098/rsbl.2021.0596

**Published:** 2022-04-13

**Authors:** Svetlana Maslakova, Christina I. Ellison, Terra C. Hiebert, Frances Conable, Maureen C. Heaphy, Dagoberto E. Venera-Pontón, Jon L. Norenburg, Megan L. Schwartz, Nicole D. Moss, Michael J. Boyle, Amy C. Driskell, Kenneth S. Macdonald, Eduardo E. Zattara, Rachel Collin

**Affiliations:** ^1^ Oregon Institute of Marine Biology, 63466 Boat Basin Road, Charleston, OR 97420, USA; ^2^ Smithsonian Tropical Research Institute, Apartado Postal 0843-03092, Balboa Ancon, Panama; ^3^ Department of Invertebrate Zoology, National Museum of Natural History, Smithsonian Institution, Washington, DC 20560, USA; ^4^ Laboratories of Analytical Biology, National Museum of Natural History, Smithsonian Institution, Washington, DC 20560, USA; ^5^ University of Washington, 1900 Commerce Avenue, Tacoma, WA 98420, USA; ^6^ INIBIOMA, Universidad Nacional del Comahue-CONICET, Bariloche, Río Negro, 8400, Argentina

**Keywords:** Nemertea, Panama, Oregon, DNA barcoding, biodiversity, planktonic larvae

## Abstract

Biodiversity assessments are critical for setting conservation priorities, understanding ecosystem function and establishing a baseline to monitor change. Surveys of marine biodiversity that rely almost entirely on sampling adult organisms underestimate diversity because they tend to be limited to habitat types and individuals that can be easily surveyed. Many marine animals have planktonic larvae that can be sampled from the water column at shallow depths. This life stage often is overlooked in surveys but can be used to relatively rapidly document diversity, especially for the many species that are rare or live cryptically as adults. Using DNA barcode data from samples of nemertean worms collected in three biogeographical regions—Northeastern Pacific, the Caribbean Sea and Eastern Tropical Pacific—we found that most species were collected as either benthic adults or planktonic larvae but seldom in both stages. Randomization tests show that this deficit of operational taxonomic units collected as both adults and larvae is extremely unlikely if larvae and adults were drawn from the same pool of species. This effect persists even in well-studied faunas. These results suggest that sampling planktonic larvae offers access to a different subset of species and thus significantly increases estimates of biodiversity compared to sampling adults alone. Spanish abstract is available in the electronic supplementary material.

## 1. Background

As much as 70–90% of marine eukaryotic species remain to be discovered and described [1,2]. This is a major impediment to identifying areas of high diversity for conservation, for understanding ecosystem function and for monitoring changes related to habitat destruction or climate. Initiatives to accelerate documenting Earth's biodiversity often apply DNA barcoding to either individual samples or to mixed samples such as gut contents, plankton tows or to environmental DNA (eDNA) samples collected directly from water or the air [3–6]. Discovery of putative species now far outpaces species identification and description, as DNA barcoding consistently reveals a large number of previously unnamed species. Adult forms are the almost exclusive focus of this approach as they are for most traditional biodiversity surveys because most metazoan species descriptions and, consequently, species identifications are based on adult morphology and because many adult forms are macroscopic. However, many adult forms are rare and many live cryptically, which makes them difficult to sample. Many marine animals also possess morphologically distinct planktonic larval stages that are spatially separated from their adults. We argue that exclusive focus on adults results in significant underestimation of diversity, which could be rectified by sampling larvae.

DNA sequence-based comparisons between adults and planktonic larvae are not new. In a pioneering study, Barber and Boyce [7] found that only 50% of the gonodactyloid stomatopod larval operational taxonomic units (OTUs) collected in the Coral Triangle could be matched to adults, despite having reference sequences for more than 90% of the known species from the region. Studies focused on planktonic stages in less well-studied regions, or of understudied groups like hemichordates, phoronids or certain families of polychaetes, report similar or greater match discrepancies, likely due to poor sampling of the adult fauna [8–12]. However, even studies that include both life stages report larvae for which the adult forms have not been detected [13,14] or were not sampled at the same site [15]. As DNA barcoding consistently reveals large numbers of previously undocumented OTUs even in relatively well-studied regions, this discrepancy between adult and larval OTUs could simply result from both larval and adult samples being relatively poor representations of the same local fauna. In this case, as sampling improves, the proportion of OTUs represented by both adults and larvae should increase and eventually approach 100% agreement in faunas where most species possess a free-living larval stage. In areas with low prevalence of planktonic development, many adults will lack a corresponding larval stage, but most of the larvae should have an adult match. An alternative hypothesis is that samples of larvae and adults are drawn from different faunas. For example, many of the larvae may belong to adults that are found in habitats that cannot be effectively sampled, like deep-water soft-bottomed environments, or are advected from a different geographical region.

**Figure 1.**
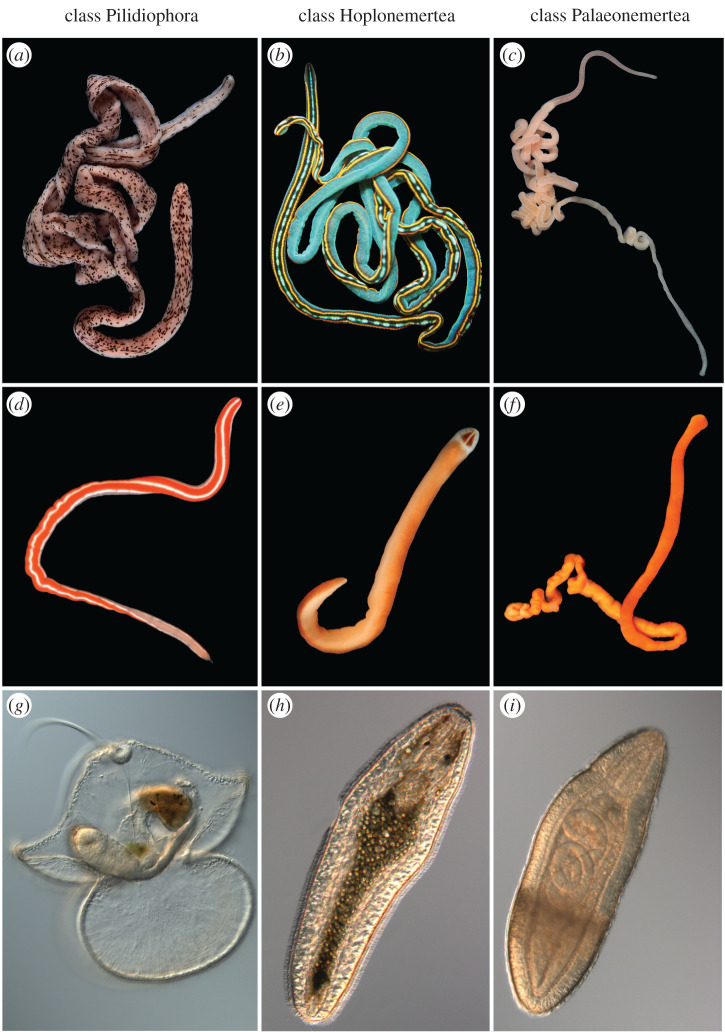
Examples of adult and larval nemerteans. (*a*) *Baseodiscus* sp. (*b*) *Tetranemertes* sp. (*c*) *Cephalothrix major*. (*d*) *Micrura* sp. (*e*) *Nipponnemertes bimaculata*. (*f*) *Tubulanus ruber*. (*g*) Pilidium larva of *Kulikovia* sp. (*h*) Planuliform larva of *Poseidonemertes collaris*. (*i*) Planuliform larva of *Tubulanus* sp.—not yet seen in its adult form. Photos by S.M., T.C.H. and C.I.E. except: (*a*) Reyn Yoshioka and (*f*) Rebecca Orr. (*a,c,e,f,g–i*) From Oregon, USA. (*b*,*d*) From Bocas del Toro, Panama.

Ribbon worms (phylum Nemertea), the focal group in this study, are important in marine systems as predators [e.g. 16–18]. About 1350 species are currently accepted [19], but many more remain unnamed [20]. Appeltans *et al*. [1] estimated that 700–1400 nemertean species are undescribed, but the rate of discovery of previously undetected or cryptic species in different parts of the world suggests that the actual diversity may be an order of magnitude larger (see §4). Although direct evidence is lacking for most nemertean species, we can infer that the majority of species in each of the three major classes—Pilidiophora Thollesson and Norenburg 2003, Palaeonemertea Hubrecht 1879 and Hoplonemertea Hubrecht 1879—have planktonic larvae, with pelagic durations of weeks to months [21]. Most pilidiophorans have a distinctive pilidium larva, while palaeonemerteans and hoplonemerteans produce juvenile-like planuliform larvae (figure 1). Here, we show how adult and larval diversity of nemerteans assayed by DNA barcoding compares in three different parts of the world and demonstrate that, even in well-sampled regions, adult and larval collections appear to represent different faunas.

## 2. Methods

We collected adult and larval nemerteans from three marine biogeographical regions between 2003 and 2020: Northeastern Pacific (Oregon, USA), the Caribbean (Bocas del Toro, Panama) and Eastern Tropical Pacific (Bay of Panama, Panama). Adults were collected intertidally and in shallow subtidal habitats (using SCUBA) either by hand or extracted from bulk collections of coral rubble, algal mats, seagrass or kelp holdfasts. Larvae were collected via plankton tows. Adults were photographed live and preserved as morphological vouchers and for DNA extraction. Larvae were photographed live and preserved whole for DNA extraction. Plankton collections in Oregon covered larvae of all three classes, while in Panama sampling focused on the pilidiophoran larvae, with only a few samples representing palaeonemerteans and hoplonemerteans. We amplified and sequenced an approximately 658 bp fragment of cytochrome c oxidase subunit 1 commonly used for DNA barcoding of metazoans [3,4,6,22]. Our analyses also include previously published sequences [23–36]. Details of collecting, laboratory methods and data analysis, GenBank accession numbers and sequence sources are available in Supplemental Information (electronic supplementary material, supplemental file 1 and table S2). Specimen details and DNA sequences are available in Barcode of Life Datasystems [37] (dx.doi.org/10.5883/DS-LARVADUL).

The dataset was exported from BOLD using MUSCLE [38] alignment, and sequences trimmed to the same length (423 bp). ABGD analysis [39] using K2P distances grouped sequences into OTUs (electronic supplementary material, table S2). Each OTU was assigned the status of ‘adult only’, ‘larval only’ or ‘mixed’. To estimate how well we had sampled each region, we constructed species accumulation curves for datasets ‘all Nemertea adults’ and ‘all Nemertea larvae’. Because non-pilidiophoran nemertean larvae were not targeted for collection in Panama, we also constructed the same curves for the Pilidiophora-only. The asymptote obtained from fitting a biexponential 5P model to the curve generated from 5000 randomized replicates of each dataset for each region was used to estimate the total number of OTUs [40,41].

We evaluated whether the observed number of mixed OTUs was different from the random expectation given the overall number of adults and larvae sampled and the numbers of individuals sampled for each OTU. We obtained frequency distributions for the number of OTUs that contain a mixture of adults and larvae separately for each of the three regions by generating 5000 permutations of each dataset, with individual adults and larvae randomly assigned to each OTU (with the same distribution of OTU sizes as the original dataset). We then compared the number of observed mixed OTUs to this distribution.

## 3. Results

**Figure 2.**
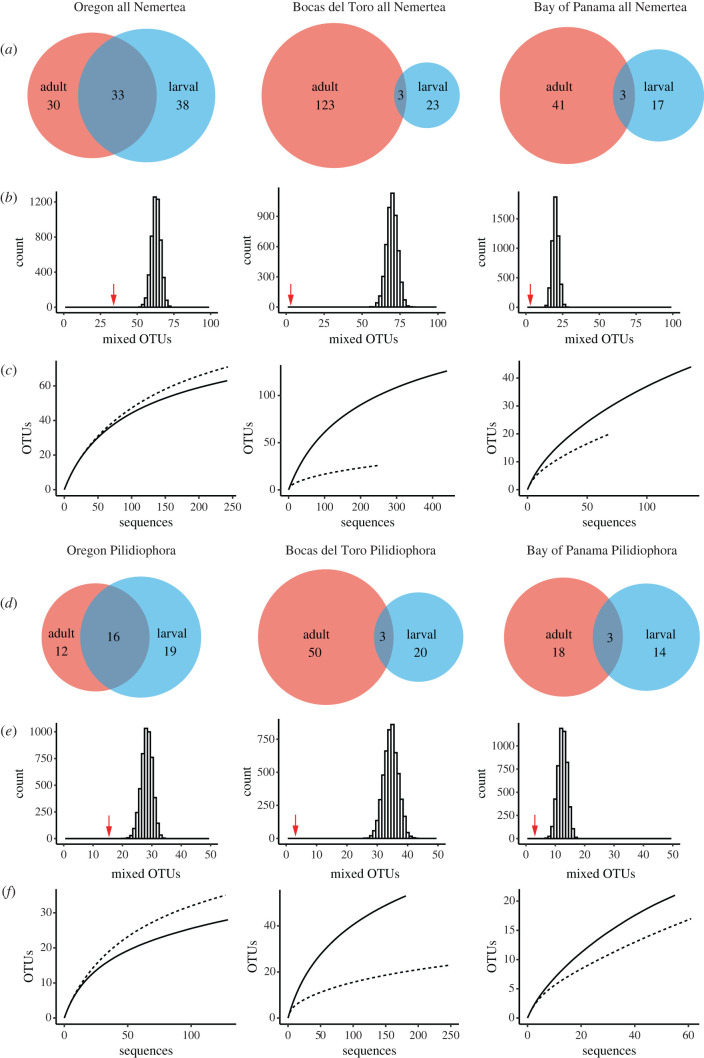
Venn diagrams showing larval-only (blue), adult-only (red) and mixed (grey) OTUs by region (*a*,*d*). Results of randomization analyses showing the expected frequency distributions of OTUs that contain a mixture of adults and larvae by region (*b*,*e*) compared to the observed number (indicated by a red arrow). Species accumulation curves for larval (dash line) and adult (solid line) faunas (*c*,*f*).

The ABGD analysis identified a clear barcoding gap between 3% and 12% K2P distances and partitioned the dataset of 1384 sequences into 308 OTUs: 101 OTUs from 485 sequences for Oregon, 149 OTUs from 693 sequences for Bocas del Toro and 61 OTUs from 206 sequences for Bay of Panama (table 1 and figure 2*a,d*; electronic supplementary material, table S1). Species accumulation curves show that the fauna of the Bay of Panama is the least well sampled, with 65% and 54% of the predicted OTUs encountered in our adult and larval collections, respectively. The Oregon and Bocas del Toro datasets included 83% and 79% of the predicted adult OTUs and 82% and 76% of the predicted larval OTUs, respectively (table 2 and figure 2*c*,*f*; electronic supplementary material, supplemental file 1).

**Table 1.** Numbers of larval and adult individuals and operational taxonomic units (OTUs) sampled from each region. Counts in parentheses exclude OTUs represented by a single individual, which cannot be mixed by definition.

	Oregon	Bocas del Toro	Bay of Panama
no. of adults sequenced	242	440	137
no. of larvae sequenced	243	253	69
total number of OTUs	101 (78)	149 (97)	61 (30)
adults only	30 (21)	123 (83)	41 (19)
larvae only	38 (24)	23 (11)	17 (8)
mixed	33	3	3
per cent mixed	34% (42%)	2% (3%)	5% (10%)
Pilidiophoran OTUs	47 (34)	73 (45)	35 (17)
adults only	12 (8)	50 (31)	18 (8)
larvae only	19 (10)	20 (11)	14 (6)
mixed	16	3	3
per cent mixed	34% (47%)	4% (7%)	9% (18%)
Hoplonemertean OTUs	32 (27)	60 (46)	20 (10)
adults only	13 (9)	59 (46)	19 (9)
larvae only	8 (7)	1 (0)	1 (1)
mixed	11	0	0
per cent mixed	34% (41%)	0% (0%)	0% (0%)
Palaeonemertean OTUs	22 (17)	16 (5)	6 (3)
adults only	5 (4)	14 (5)	4 (2)
larvae only	11 (7)	2 (0)	2 (1)
mixed	6	0	0
per cent mixed	27% (35%)	0% (0%)	0% (0%)

**Table 2.** Results of the rarefaction analysis by region for all Nemertea and Pilidiophora only. See electronic supplementary material (table S1 in supplemental file 1) for an expanded version, including other estimators and predicted sample sizes.

	Oregon all Nemertea		Bocas del Toro all Nemertea		Bay of Panama all Nemertea	
	adult	larval	adult	larval	adult	larval
OTUs found	63	71	126	26	44	20
OTUs estimated	75.8	86.7	157.2	34.2	66.8	37.7
lower 95%	75.5	86.5	157	33.8	66.5	37.2
upper 95%	76.1	86.9	157.4	34.3	67.0	38.3
sample size	242	243	440	253	137	69

	Oregon Pilidiophora		Bocas del Toro Pilidiophora		Bay of Panama Pilidiophora	
	adult	larval	adult	larval	adult	larval
OTUs found	28	35	53	23	21	17
OTUs estimated	35.0	42.9	69.8	28	31.8	38.9
lower 95%	34.7	42.6	69.5	27.8	31.6	38.1
upper 95%	35.4	43.3	70.1	28.2	31.9	39.8
sample size	129	127	181	250	55	61

The percentages of OTUs that were mixed (i.e. included both adult and larval samples) were small: 33% for Oregon, 2% for Bocas del Toro and 5% for Bay of Panama (table 1 and figure 2*a*). Because larval sampling efforts in Panama focused primarily on pilidium larvae, and few larvae of the other two classes were collected, we recalculated this for Pilidiophora-only and found 34%, 4% and 9% of the OTUs were mixed in Oregon, Bocas del Toro and Bay of Panama, respectively (table 1 and figure 2*d*). When singleton OTUs, which by definition cannot be mixed, were excluded, the mixed OTUs represented 42%, 3% and 10% for the three classes combined, and 47%, 7% and 18% for the Pilidiophora-only in Oregon, Bocas del Toro and Bay of Panama, respectively (table 1).

Randomization tests showed that for all three faunas, the number of OTUs with a mix of larvae and adults was significantly smaller than would be expected if larval and adult samples were assigned to OTUs at random. In fact, the observed number of mixed OTUs (indicated by red arrows on figure 2*b*,*e*) was well outside the distributions generated by the randomization from all three sites and for both the total nemertean dataset and the Pilidiophora-only, showing that adult and larval samples are not drawn from the same species pool.

## 4. Discussion

The scope of the challenge faced by biologists working to document marine biodiversity defies comprehension. The oceans are vast, the most diverse sites are remote and difficult to sample and very few researchers are engaged in this endeavour. Nemertean worms are macroscopic and ecologically important as predators, yet over the last century only a handful of taxonomy experts have been active in documenting nemertean species at any one time. It is no surprise then that with relatively intensive sampling at three geographical regions, we documented 308 OTUs, nearly 25% of the number of currently accepted nemertean species names [19]. Notably, greater than 90% of these are either undescribed or part of cryptic species complexes that include undescribed species. What is surprising, however, is that approximately 25% are represented only by larvae (i.e. no corresponding adults were sequenced). The percentage varies somewhat by region, but it is always a large fraction regardless of the sampling intensity. In the Oregon fauna, which has been the focus of intense collecting and study for the last 13+ years [21,26,27,30–32,35,36,42–44], and for which we estimate approximately 80% of the fauna has been sampled, 38% of all OTUs and 40% of pilidiophoran OTUs are known only in larval form. The proportion is slightly less in Panama, where sampling efforts have been less intensive. On the Caribbean coast (Bocas del Toro), where larvae are less well-sampled than adults, 15% of all OTUs and 27% of pilidiophoran OTUs are larval-only. On the Pacific coast (Bay of Panama) where sampling has been very limited for both larvae and adults, 28% of all OTUs and 40% of pilidiophoran OTUs are known only from larvae. This presents a compelling case that sampling larvae, regardless of overall sampling intensity, can significantly increase the documented diversity.

This is not the first study to find larval or juvenile OTUs that cannot be matched to local adults, but it is one with the largest sample size, both in terms of the number of OTUs and the number of specimens. The apparent mismatch between larval and adult samples is not limited to nemerteans or even to marine taxa [7,14,45–50]. What is the cause of this pattern? It is likely that our plankton samples include the larvae of animals for which adults are difficult to collect. For example, in Bocas del Toro, we collected adults primarily from coral rubble down to 15 m depth. However, most of the Almirante Bay is comprised of a slightly deeper (20 m) mud bottom, which almost certainly harbours a distinct fauna compared to the reefs, but is substantially more challenging to sample directly. It is likely that larval faunas include some unknowable proportion of species that have been advected into the area and do not occur locally as adults, but even such advected larvae, nevertheless, are functional participants in the ecosystem.

Finally, if an appreciable number of species can be detected in the water column, could eDNA be an effective approach to assess local diversity? Some recent studies suggest that an eDNA approach is of limited use [51]. We compared our data to that of Nguyen *etal*. [52] who analysed eDNA samples taken from 134 sites throughout Almirante Bay, targeting areas with known coral, seagrass, mangrove, sandy and artificial substrates. Although nemerteans had the 20th highest read abundance among the major groups of metazoans and other eukaryotes sequenced, only 26 of the 8586 OTUs were identified as Nemertea. Fifteen of these 26 appear to be misidentified at the phylum level (see electronic supplementary material, supplemental file 1 for criteria). The 11 OTUs that do appear to be Nemertea are a tiny fraction of the 149 OTUs we report. However, six of these 11 do represent new diversity (OTUs we did not detect). These results suggest that although eDNA can detect diversity in a wide array of organisms quickly from a large number of samples, targeted sampling of focal taxa including both adult and larval samples remains the most effective way to document undiscovered diversity.

## Data Availability

Sequence data and specimen details, including collecting information, can be accessed through the Barcode of Life Database https://doi.org/10.5883/DS-LARVADUL [37]. Taxonomic information, OTU composition, GenBank accession, BOLD ID numbers, life stage, region, and sources for each sequence can be found in the electronic supplementary material (table S2). Details of methods (collecting, specimen processing, molecular work, data analysis) can be found in the electronic supplementary material (supplemental file 1).
